# Role of Interleukin(IL)-6 in NK Activity to Hypoxic-Induced Highly Invasive Hepatocellular Carcinoma(HCC) Cells

**DOI:** 10.4014/jmb.2304.04023

**Published:** 2023-06-12

**Authors:** Hwan Hee Lee, Hyojung Kang, Hyosun Cho

**Affiliations:** 1 Department of Pharmacy, Duksung Women’s University, Seoul 01369, Republic of Korea; 2 Duksung Innovative Drug Center, Duksung Women’s University, Seoul 01369, Republic of Korea; 3 Vessel-Organ Interaction Research Center, VOICE (MRC), Cancer Research Institute, College of Pharmacy, Kyungpook National University, Daegu 41566, Republic of Korea

**Keywords:** Natural killer (NK) cell, Hepatocellular carcinoma (HCC), hypoxia, HIF-1α, Interleukin (IL)-6

## Abstract

Natural killer (NK) cell dysfunctions against hepatocellular carcinoma (HCC) in a hypoxic environment. Many solid tumors are present in a hypoxic condition, which changes the effector function of various immune cells. The transcription of hypoxic-inducible factors (HIFs) in cancer cells make it possible to adapt to their hypoxic environment and to escape the immune surveillance of NK cells. Recently, the correlation between the transcription of HIF-1α and pro-inflammatory cytokines has been reported. Interleukin (IL)-6 is higher in cancers with a highly invasive ability, and is closely related to the metastasis of cancers. This study showed that the expression of HIF-1α in HCC cells was associated with the presence of IL-6 in the environment of HCC-NK cells. Blocking of IL-6 by antibody in the HCC-NK interaction changed the production of several cytokines including TGF-β, IL-1, IL-18 and IL-21. Interestingly, in a co-culture of HIF-1α-expressed HCC cells and NK cells, blocking of IL-6 increased the production of IL-21 in their supernatants. In addition, the absence of IL-6 significantly enhanced the cytotoxic ability and the expression of the activating receptors (NKG2D, NKp44, and NKG2C) in NK cells to HIF-1α-expressed HCC cells. These effects might be made by the decreased expression of HIF-1α in HCC cells through the inhibited phosphorylation of STAT3. In conclusion, the absence of IL-6 in the interaction of HIF-1α-expressed HCC cells and NK cells could enhance the antitumor activity of NK cells to HCC cells.

## Introduction

Pro-inflammatory cytokines in a tumor microenvironment (TME) are an important factor in the surveillance of tumors by various immune cells [1]. Recent studies have shown that pro-inflammatory cytokines such as interleukin (IL)-1β, tumor necrosis factor (TNF)-α, and IL-6 in a tumor microenvironment contribute to the development and metastasis of cancers through the immune escape of cells [2-4]. Of these, IL-6 is known to be a cytokine that leads to chronic inflammation in several cancers [5, 6]. According to a meta-analysis, the level of IL-6 in the serum of HCC patients was higher than healthy controls [7], and in particular, it is highest in stage III compared to stage I or II [8].

A natural killer (NK) cell is an innate immune lymphocyte that plays an important role in cancer development. NK cells can directly kill cancer cells and help other immune cells indirectly through the production of several cytokines such as IFN-γ [9, 10]. NK cells account for about 55% of hepatic lymphocytes, and are in the first line of defense of the liver against pathogens [11]. The fate of pathogens in elimination by the host is decided by the expression of receptors on the surface of NK cells. The superiority of activating receptors such as c-lectin-like receptors NKG2D, NKG2C and NKG2E, natural cytotoxicity receptors (NCRs) NKp30, NKp44 and NKp46 and killer cell immunoglobulin-like receptors (KIRs) KIR-2DS and KIR-3DS on the surface of NK cells increase the antitumor activity of NK cells [12]. They are influenced by various factors represented in the tumor microenvironment. For instance, a high level of transforming growth factor (TGF)-β in the sera of glioblastoma patients induces the downregulation of NKG2D receptors on the surface of NK cells and CD8^+^ T cells [13]. NK cells primed by IL-1-related pro-inflammatory cytokine IL-18 can promote antitumor immunity by increasing the production of Th1- and CTL-driving IL-12 in a human ovarian cancer environment [14].

Hypoxia is a property that constitutes the tumor microenvironment, and is present in many solid tumors. The cancer cells within the hypoxic regions of a solid tumor undergo a change of metabolic system to adapt to the low oxygen environment. Thus, they develop a survival system and overcome death. The transcription of hypoxia-inducible factors (HIFs) is increased in many tumors and is a regulator in oxygen homeostasis. The expression of HIF-1α in human colon cancer cells downregulates pro-apoptotic proteins such as the Bcl-2 family [15]. In addition, the increased expression of HIF-1α in breast cancer cells induces the transcription of the survivin gene related to survival signals under normoxia (O_2_ < 20%) but normal mammary epithelial cells are not in it [16]. Resistance to apoptosis or genomic instability in tumor cells can be induced by a dysfunction of p53 and the DNA repair system in hypoxic tumors [17, 18]. The enhanced survivability of cancer cells under hypoxia might generate an immune escape. Recent studies have shown that the transcription of hypoxic-related protein HIFs in tumors impairs the effector function of NK cells or cytotoxic T cells [19, 20]. A major histocompatibility complex class I chain-related (MIC) that binds to activating receptor on the surface of NK cell is shed by the transcription of HIFs in pancreatic carcinoma [20]. Moreover, the recent studies have shown a correlation between the expression of HIF-1α in tumors and the presence of cytokines in the tumor microenvironment. The differentiation of Th17 cells is increased through the expression of HIF-1α by IL-6 and TGF-β collaboratively [21, 22] and they can promote tumor growth through angiogenic and immunosuppressive effector functions [23, 24]. In this regard, we hypothesized that the pro-inflammatory cytokines in the interaction of highly invasive HCC cells and NK cells could influence the expression of HIF-1α in cancer cells and that lead to the immune escape of NK cells.

## Materials and Methods

### Reagents

Cobalt chloride (CoCl_2_) is commonly used for HIF-1α expression and was purchased from Sigma Aldrich Inc.(USA), and human IL-6 antibody and recombinant IL-21 were obtained from BioLegend Inc. (USA). CoCl_2_ was dissolved with sterile water and diluted in complete media. Cells were treated after all reagents were filtered through a 0.2 μm pore size.

### Cell Culture and Treatment

Human hepatocellular carcinoma (HCC) cell lines SK-Hep1 and Hep3B, and natural killer (NK) cell line NK-92 were distributed by American Type Culture Collection (ATCC, USA). SK-Hep1 and Hep3B cells were incubated in Dulbecco’s Modified Eagle Medium (DMEM; Gibco, USA and Hyclone, USA, respectively) with 10% heat-inactivated FBS (Gibco), and NK-92 were incubated in alpha-MEM (Gibco) supplied 20% FBS (Gibco) and human recombinant (hr) IL-2 (200 U/ml, BioLegend). All media were included with 100 U/ml penicillin and streptomycin (Gibco) and filtered through a 0.45 μm pore size. In the state of a co-culture of HCC and NK-92, CoCl_2_ was treated for 24 h to express HIF-1α. Human IL-6 antibody (2 ng/ml) or hrIL-21 (1 ng/ml) were added to media according to the purpose.

### Analysis of Cytokines Using ELISA Assay

The quantitation of cytokines was analyzed by BD OptiEIA Set Human IL-1β and TGF-β1 (BD Bioscience), and Human ELISA Kit IL-18 and IL-21 (Invitrogen, USA). Briefly, target cells were culutred at a density of 5 × 10^5^ cells per well of a 6-well plate overnight (18-24 h), and co-cultured with effector cells (NK-92) at a ratio of 2:1 (E:T) for 24 h with or without IL-6 antibody (2 ng/ml) or hrIL-21 (1 ng/ml). The supernatants co-cultured were harvested and stocked at -80°C before experiments. All experiments were performed in accordance with manufacturer’s instructions. The absorbances were measured at 450 nm with λ correction on 570 nm or 620 nm using a microplate reader (BMG Labtech, Germany).

### The Cytotoxic Effect of NK-92 Cells on Target Cells Using LDH-Release Assay

NK cytotoxicity to target cells was analyzed by LDH-release assay (CytoTox96 Non-Radioactive Cytotoxicity Assay Kit, Promega, USA). Briefly, target cells were incubated at a density of 5 × 10^3^ cells each in a 96-well flat bottom plate overnight (18-24 h), and co-cultured with effector cells (NK-92) at a ratio of 2:1 (E:T) with or without CoCl_2_ (250 μM) or IL-6 antibody (2 ng/ml) for 24 h. After co-culture, the cells were centrifuged and their supernatants were transferred to each well of a new plate. Then, CytoTox96 reagent was treated for 30 minutes, and following reaction, the absorbance was measured at 490 nm within 1 h after stop solution was treated using a microplate reader (BMG Labtech).

### Analysis of mRNA by Reverse Transcription (RT) Quantitative PCR

The level of mRNA expression in cells was assessed by reverse transcription (RT) quantitative PCR. Briefly, target cells were co-cultured effector cells (NK-92) with or without the treatment of CoCl_2_ (250 μM) or IL-6 antibody (2 ng/ml) for 24 h. Isolation of total RNA from cells was performed with a TaKaRa MiniBest Universal RNA Extraction Kit (Takara-Bio, Japan), and synthesis of cDNA was conducted using the PrimeScript First-strand cDNA Synthesis Kit (Taraka-Bio). Then the quantitation of mRNA was analyzed using SYBR Green dye (BioLine, UK). All experiments followed the manufacturer’s instructions. Primer sequences: Granzyme-B: F 5’-GATCATCGGGGGACATGAGG-3’, R 5’-GGTCGGCTCCTGTTCTTTGA-3’; Perforin: F 5’-TCCTAAGCC CACCAGCAATG-3’, R 5’-CCCCATGCTTGGATGAAGGT-3’; GAPDH: F 5’-CACACCATCTTCCAGGAGC-3’, R 5’-CATGAGTCCTTCCACGATACC-3’. Conditions for RT-qPCR: 20 s at 94°C for denaturation, 20 s at 60°C for annealing, and 1 min at 72°C for extension for 40 cycles and melting cycle.

### Receptors on the Surface of NK-92 Using Fluorescence Antibodies

Proteins on the surface of NK-92 cells were analyzed by surface staining with fluorescence antibodies. Briefly, the effector (NK-92) cells co-cultured with target cells with or without the treatment of anti-IL-6 (2 ng/ml) for 24 h were stained with anti-CD94-PE (BD Biosciences), anti-CD178-PE (BD Biosciences), anti-NKG2D-PE (BD Biosciences), anti-NKG2C-PE (BD Biosciences), anti-NKp30-PE (BD Biosciences), anti-NKp44-PE (BD Biosciences), anti-CD56-APC (BD Biosciences) and VP for 30 minutes. Then the stained cells were detected using flow cytometry (Novocyte, Flow Cytometer, ACEA Biosciences, USA).

### Western Blot Analysis

The expression of proteins was investigated by Western blot analysis. Briefly, proteins were extracted from cells with protein extraction buffer (Intron, Korea) with protease and phosphatase inhibitor (Gendepot, USA) and then the extracted proteins were quantified using the Bradford (Coomassie blue) assay (Gendepot). The quantified protein was separated by electrophoresis, transferred to a polyvinylidene fluoride (PVFD) microporous membrane (Millipore, USA), and blotted with first antibodies for at least 18 h. The first antibody-blotted membranes were blotted with secondary antibodies for 1 h, and then were visualized in an enhanced chemiluminescent detection solution under Chemi-doc (Millipore). The first antibodies were as follows: HIF-1α (Cell Signaling, USA), granzyme-B (Santa Cruz Biotechnology, USA), (p)-Stat3 (Cell Signaling), (cleaved)-caspases-3, 7, 8 and 9; cell signaling), Fas (CD95; Santa Cruz Biotechnology), FADD (Santa Cruz Biotechnology), and GAPDH (Santa Cruz Biotechnology).

### Statistical Analyses

All experimental results were analyzed by Microsoft Excel. The data were conducted at least three times for presenting as mean ± SD. Several means were compared as one-way or two-way analysis of variance followed by Fisher’s exact test. Differences between groups were considered significant at a *p*-value of less than 0.05.

## Results

### Change of Cytokines Secreted from the Co-Culture of HCC SK-Hep1 Cells and NK-92 Cells by Influence of IL-6

**Fig. 1:**
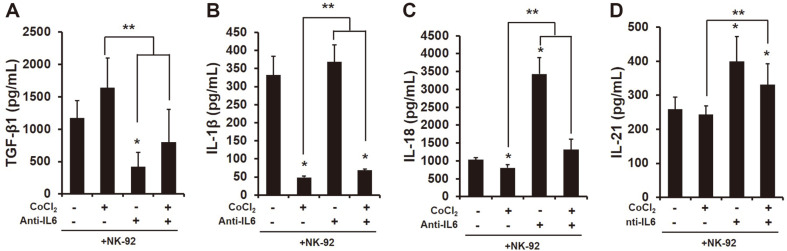
Change of cytokines released from the co-culture of HIF-1α-induced HCC SK-Hep1 and NK-92. The quantitation of TGF-β1 (**A**), IL-1β (**B**), IL-18 (**C**), and IL-21 (**D**) released from the co-culture of HCC SK-Hep1 and NK-92 with or without CoCl_2_ (250 μM) and IL-6 antibody (2 ng/ml) by ELISA assay. *p < 0.05 is versus non-treated; **p < 0.05 is versus CoCl_2_-treated. All data are presented as the means ± SD in three independent experiments.

Several studies have shown the difference between cytokines produced by high and low invasive cancers [25]. In a previous study, pro-inflammatory cytokine IL-6 was higher in a highly invasive breast cancer cell line MDA-MB-231 than a low invasive cancer cell line MCF-7 [6]. Thus, we first examined the composition secreted from the co-culture of HCC cells and NK-92 cells. The results indicated that pro-inflammatory cytokines including IL-1β and IL-6 were significantly higher in the co-culture of highly invasive HCC cell lines SK-Hep1 and NK-92 than low invasive HCC cell line Hep3B (Fig. S1). In addition, there was no change in pro-inflammatory cytokines in the induction of HIF-1α expression by the treatment of CoCl_2_ (250 μM) (Fig. S2A). However, in the co-culture of NK-92 cells and SK-Hep1 cells treated with CoCl_2_ (250 μM), the production of IL-1β and IL-18 was significantly decreased (Figs. 1B and 1C). Next, we examined the composition of supernatants of the co-culture of HCC SK-Hep1 and NK-92 treated with IL-6 antibody (2 ng/ml). Interestingly, the treatment of IL-6 antibody significantly reduced the level of TGF-β1 (Fig. 1A), and produced a tremendous amount of IL-18 and IL-21 (Figs. 1C and 1D) in their supernatants. In addition, the production of IL-21 was greatly maintained in the treatment of IL-6 antibody with CoCl_2_ similar to the treatment of IL-6 antibody (Fig. 1D).

### Increase in NK Cytotoxicity to HCC, and High Production of Granules (gzy-B and perforin) in NK Cells Expressing HIF-1α through the Blockade of IL-6

**Fig. 2:**
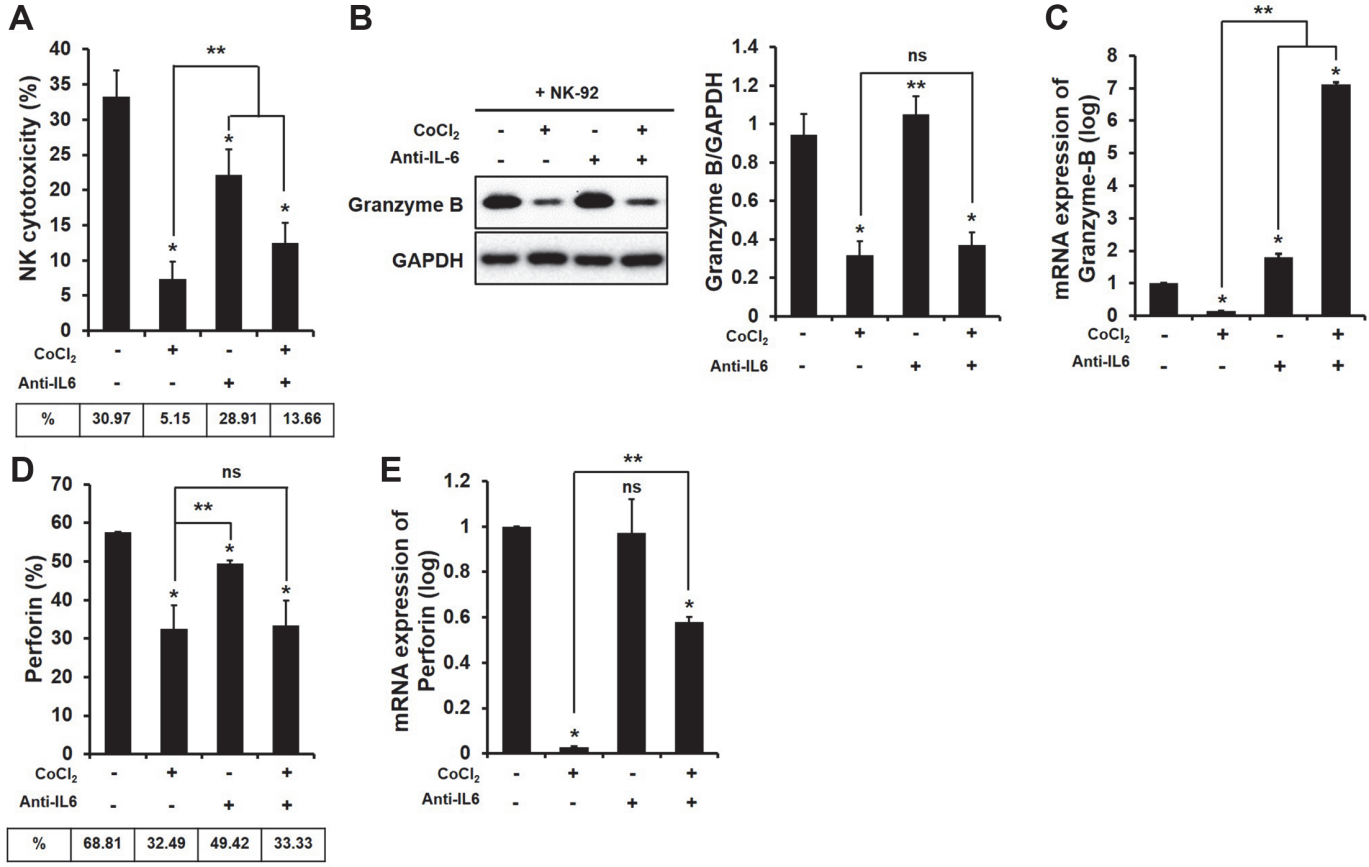
Increase in NK cytotoxicity and granules in the coculture of NK-92 and HCC SK-Hep1 with HIF-1α expression by the blocking of IL-6. (**A**) Cytotoxic ability of NK-92 co-cultured with HCC SK-Hep1 with or without the treatment of CoCl_2_ (250 μM) or IL-6 antibody by LDH-release assay. (**B, D**) Granules (Grz-B and perforin) in NK-92 cells cocultured with HCC SK-Hep1 treated with or without CoCl_2_ (250 μM) or IL-6 antibody. The expression of Grz-B and perforin was measured by Western blot analysis and intracellular staining with fluorescent perforin antibody, respectively. The expression of mRNA of (**C**) grz-B and (**E**) perforin in NK-92 co-cultured with HCC SK-Hep1 is analyzed using RT-qPCR. All experiments were conducted in three independent as means ± SD. *p < 0.05 vs. non-treated; **p < 0.05 vs. CoCl_2_.

**Fig. 3:**
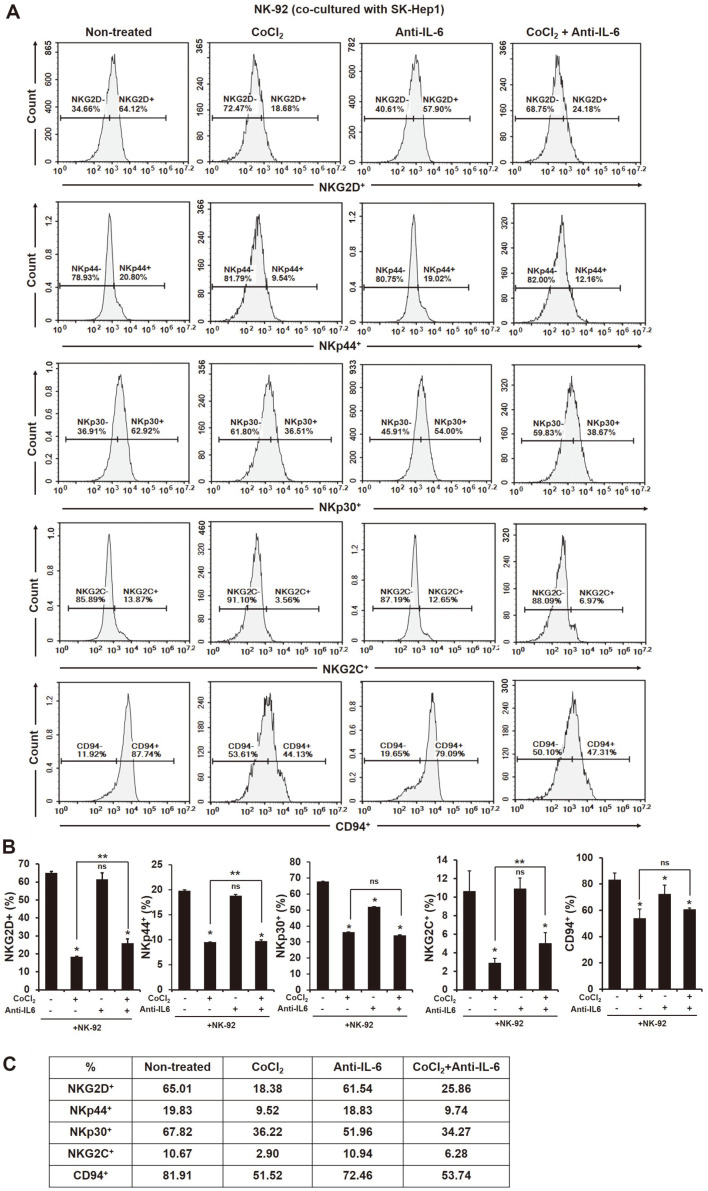
The expression of activating receptors on the surface of NK-92 co-cultured with HCC SK-Hep1. The effector cells were co-cultured with target cells at a ratio of 2:1 (E:T) for 24 h. After co-culture, the effector cells were stained with fluorescent antibodies and analyzed using flow cytometry. (**A**) Representative histograms of NKG2D, NKp44, NKp30, NKG2C, and CD94, (**B**) the graphs of their positive population, and (**C**) the quantification table on the average of three independent experiments. *p < 0.05 vs. non-treated; **p < 0.05 vs. CoCl_2_.

The level of cytokines produced by the co-culture of HCC SK-Hep1 and NK-92 was changed as shown in Fig. 1. Subsequently, we investigated the cytotoxicity and the granules (gzy-B and perforin) of NK-92 to HCC SK-Hep1 by the treatment of IL-6 antibody. The cytotoxic effect of NK-92 on HCC significantly decreased by the blocking of IL-6 (Fig. 2A, Non-treated 30.97%, Anti-IL-6 28.91%, *p < 0.05) however, to HIF-1α-expressed HCC cells, it remarkably enhanced compared as the non-blocking of IL-6 (Fig. 2A, Anti-IL-6 5.15%, CoCl_2_ + Anti-IL-6 13.66%, **p < 0.05). The amount of intracellular perforin of NK cells cocultured with HCC cells was significantly reduced by the blocking of IL-6 (Fig. 2B, Non-treated 68.81%, Anti-IL-6 49.42%, *p < 0.05) but mRNA of perforin was no difference (Fig. 3C, ns). Contrary to perforin, the expression of granzyme in NK cells was significantly increased by the blocking of IL-6 regardless of HIF-1α expression (Figs. 2D and 2E, *,**p < 0.05).

### Induction of NK Activating Receptors on the Surface of NK-92 Cells Co-Cultured with HIF-1α-Expressed HCC SK-Hep1 Cells by the Blocking of IL-6

NK cells express various activating or inhibitory receptors and a balance of these receptors decide NK functions on cancers [26]. As shown in Fig. 3, the expression of activating receptors on the surface of NK cells was all significantly downregulated by the treatment of CoCl_2_ compared to non-treated (Fig. 3, NKG2D^+^; non-treated 65.01%, CoCl_2_ 18.38%, NKp44^+^; non-treated 19.83%, CoCl_2_ 9.52%, NKp30^+^; non-treated 67.83%, CoCl_2_ 36.33%; NKG2C^+^; non-treated 10.67%, CoCl_2_ 2.90%, CD94^+^; non-treated 81.91%, CoCl_2_ 51.52%, *p < 0.05). In addition, the blocking of IL-6 in the supernatant of the co-culture of NK-92 cells and HCC SK-Hep1 cells downregulated the expression of NKp30 and CD94 receptors on the surface of NK-92 cells (Fig. 3, NKp30^+^; non-treated 67.82%, anti-IL-6 51.96%, CD94^+^; non-treated 81.91%, anti-IL-6 72.46%, *p < 0.05). However, the blocking of IL-6 in their supernatants significantly enhanced the expression of NK activating receptors NKG2D and NKG2C on the surface of NK-92 cells on HIF-1α-expressed HCC SK-Hep1 cells (Fig. 3, NKG2D^+^; CoCl_2_ 18.38%, CoCl_2_ + Anti-IL-6 25.86%, NKG2C^+^; CoCl_2_ 2.90%, CoCl_2_ + Anti-IL-6 6.28%, **p < 0.05).

### Induction of Apoptosis in HCC SK-Hep1 Cells Co-Cultured with NK-92 Cells by the Blocking of IL-6 in Their Supernatants

**Fig. 4:**
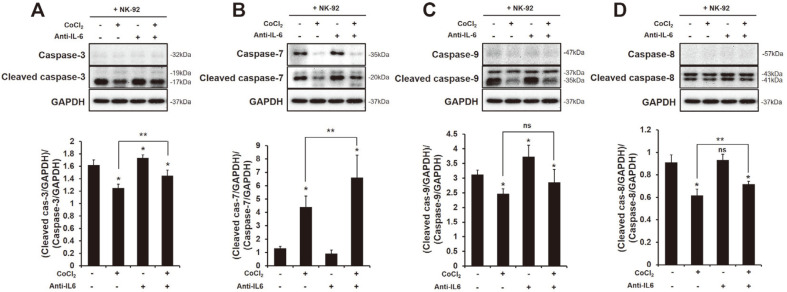
The expression of apoptotic molecules in HCC SK-Hep1 co-cultured with NK-92. Target cells were incubated in a 6-well plate overnight and then co-cultured with effector cells (NK-92) at a ratio of 2:1 (E:T) for 24 h with or without CoCl_2_ (250 μM) or IL-6 antibody (2 ng/ml). After removing the effector cells, target cells were harvested and lysed using protein extraction buffer. The extracted proteins were analyzed using Western blot analysis. The expression of (**A**) (cleaved or) caspase-3, (**B**) (cleaved or) caspase-7, (**C**) (cleaved or) caspase-9, and (**D**) (cleaved or) caspase-8 in HCC SK-Hep1 co-cultured with NK-92. *,**p < 0.05. The quantitation of bands was analyzed in three independent experiments.

The cytotoxic effect of NK-92 cells on HIF-1α-expressed HCC SK-Hep1 cells was improved by blocking sIL-6 in their supernatants by IL-6 antibody (Fig. 2A), and the activating receptor NKG2D and NKG2C were highly upregulated (Fig. 3). Therefore, we investigated whether NK activity actually generated the apoptotic signals in HIF-1α-expressed HCC SK-Hep1 cells by the blocking of IL-6. As shown in Fig. 4. the cleavage of caspase-3 and -9 in HCC SK-Hep1 cells co-cultured with NK-92 cells was significantly increased by the blocking of sIL-6 (*p < 0.05). The expression of HIF-1α in HCC SK-Hep1 cells co-cultured with NK-92 cells decreased the expression of apoptotic molecules in cancer cells. Interestingly, a decreased expression of the cleaved caspases (-3,-9 and -8) in HCC SK-Hep1 cells by HIF-1α expression was increased by the blocking of IL-6 (Figs. 3A and 3C), but wasn’t significant in cleaved caspase-9 (Fig. 3D). The apoptotic activation can be generated through two different signaling pathways [27]. The activation of caspase-8 can be induced by the interaction of death signal Fas and Fas ligand (FasL). Therefore, we examined the expression of Fas and FasL in each cell. The expression of FasL (CD178) on the surface of NK-92 cells co-cultured with HCC SK-Hep1 cells was increased by the blocking of IL-6 compared to non-blocking (Fig. S3A, Non-treated 10.31%, Anti-IL-6 14.87%, *p < 0.05), in addition, it was significantly restored to HIF-1α-expressed HCC cells (CoCl_2_ 6.84%, CoCl_2_ + Anti-IL-6 8.65%). The expression of FADD in HCC SK-Hep1 cells co-cultured with NK-92 cells was increased by the blocking of IL-6 regardless of HIF-1α expression (Fig. S3B).

### Inhibited Expression HIF-1α and pStat3 Proteins in HCC SK-Hep1 Cells Co-Cultured with NK-92 Cells by the Blocking of IL-6

**Fig. 5:**
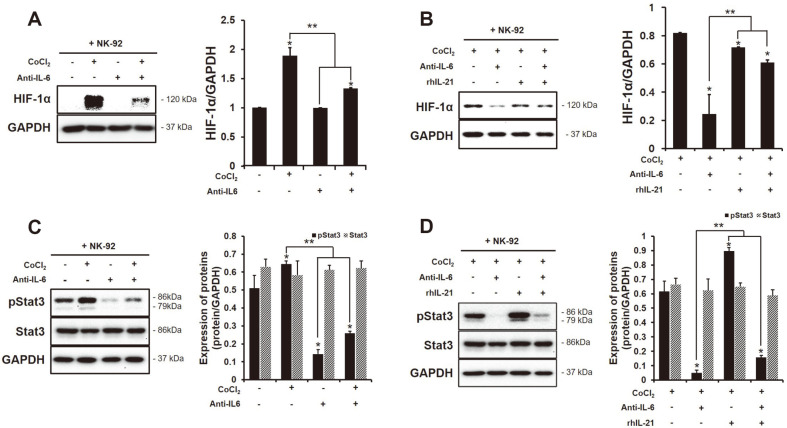
The inhibited expression of HIF-1α in HCC SK-Hep1 cells through the decrease of Stat3 expression by the blocking of IL-6. The expression of HIF-1α and (p)Stat3 was analyzed using Western blot analysis. The expression of (**A, B**) HIF-1α and (**C, D**) (p)Stat3 in HCC SK-Hep1 cells co-cultured with NK-92 cells with or without the treatment of CoCl_2_ (250 μM) or IL-6 antibody (2 ng/ml), and hrIL-21. *,**p < 0.05. All data were presented as the means ± SD in three independent experiments.

A recent study shows that *in vivo* or in vitro IL-6 is associated with the transcriptional activity of HIF-1α [28]. Both human highly invasive HCC cell lines SK-Hep1 and low invasive Hep3B could not express HIF-1α autonomously (Fig. S2A). Therefore, the expression of HIF-1α in HCC cell lines was induced by CoCl_2_ (250 μM) in a time-dependent manner. HIF-1α in human highly invasive HCC SK-Hep1 cells was continuously expressed after 6 h, whereas that in Hep3B was the highest at 6 h and decreased afterward (Fig. S2A). The expression of pStat3 in HCC SK-Hep1 cells co-cultured with NK-92 cells was more increased by the treatment of CoCl_2_ than non-treated but that was suppressed by a treatment of IL-6 antibody (Fig. 5C). The phosphorylation of Stat3 in NK-92 cells wasn’t increased with CoCl_2_ (Fig. S4A).

### Recovery of the Activating Receptors on the Surface of NK Cells Co-Cultured with HIF-1α-Expressed HCC SK-Hep1 Cells in the Presence of IL-21

**Fig. 6:**
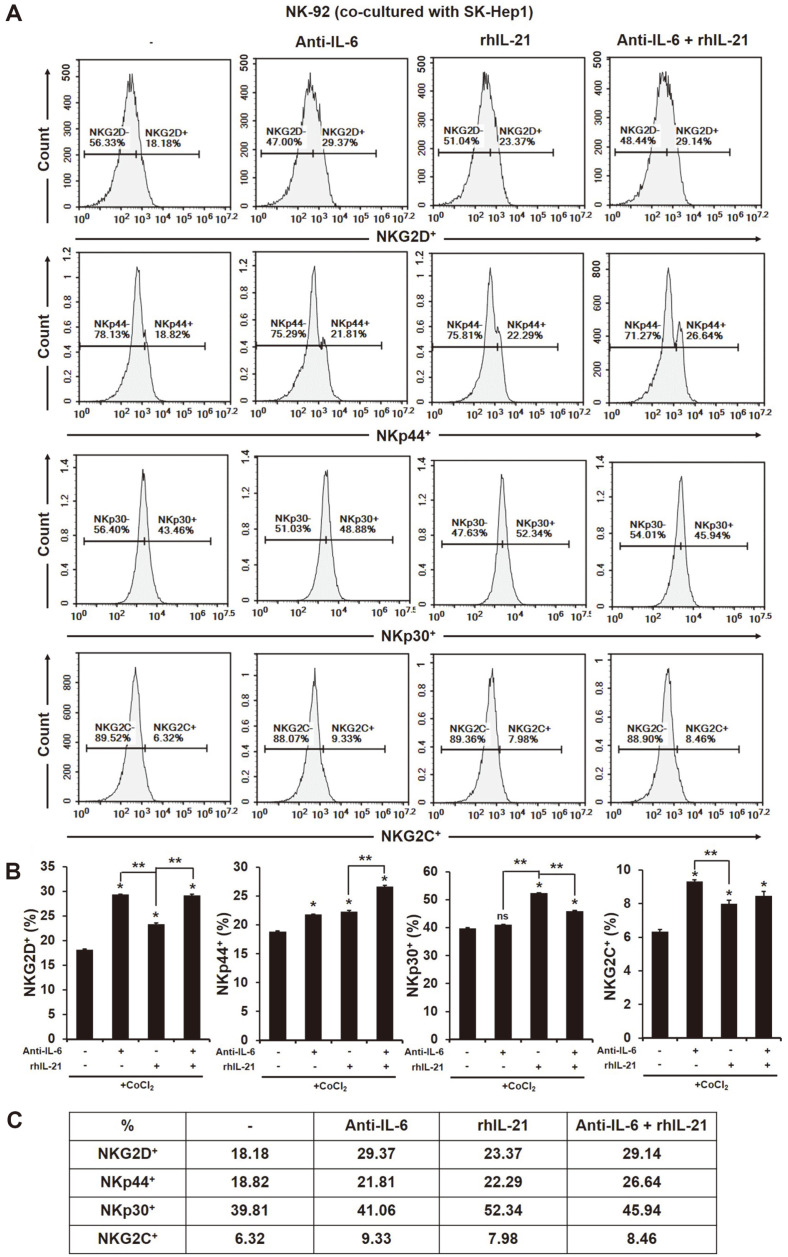
Increased expression of the activating receptors on the surface of NK cells co-cultured with HIF-1α-expressed HCC cells by the treatment of rhIL-21. The effector cells were co-cultured with target cells with the treatment of Anti-IL-6 (2 ng/ml) or rhIL-21 (1 ng/ml) at a ratio of 2:1 (E:T) for 24 h. After 24 h, the effector cells were harvested, stained with fluorescent antibodies and analyzed using flow cytometry. (**A**) Representative histograms of activating receptors, (**B**) the graphs of the positive population, and (**C**) the quantification table as the means in three independent experiments. *p < 0.05 vs. control; **p < 0.05 vs. rhIL-21.

In the co-culture of HCC SK-Hep1 cells and NK-92 cells, the blocking of IL-6 greatly increased the production of IL-21 in their supernatants (Fig. 1F). It is known that IL-21 induces the proliferation and the function of NK cell by binding the high-affinity receptor complex (a heterodimer of IL-21R and the γc) and subsequent, that activates the JAK/STAT3 pathway [29]. The activating receptors on the surface of NK cells co-cultured with HCC cells was significantly increased by the treatment of rhIL-21 (Fig. 6, NKG2D^+^; control 18.18%, rhIL-21 23.37%, NKp44^+^; control 18.82%, rhIL-21 22.29%, NKp30^+^; 39.81%, 52.34%, NKG2C^+^; control 6.32%, rhIL-21 7.98%, *p < 0.05). We expected that the treatment of rhIL-21 in the co-culture of HIF-1α-expressed HCC SK-Hep1 cells and NK-92 cells could enhance the effector function of NK cells against cancer cells compared to the treatment of IL-6 antibody alone. However, unlike our expectation, the combination with IL-6 antibody didn’t greatly increase the activating receptors on the surface of NK cells except to NKp44 (Fig. 6, NKG2D^+^; Anti-IL-6 29.37%, Anti-IL-6 + rhIL-21 29.14%, NKp44^+^; Anti-IL-6 21.81%, rhIL-21 22.29%, NKp30^+^; Anti-IL-6 41.06%, Anti-IL-6 + rhIL-21 45.94%, NKG2C^+^; Anti-IL-6 9.33%, Anti-IL-6 + rhIL-21 8.46%, **p < 0.05). NKp30 on the surface of NK cells co-cultured with HIF-1α-expressed HCC SK-Hep1 cells was highly expressed by the treatment of rhIL-21 alone compared to others (NKp30^+^; control rhIL-21 52.34%). The IL-21 signal in NK cells activates their downstream proteins such as STAT1 and STAT3, which promotes the production of granules and IFN-γ, and the cytotoxic activity [30]. The treatment of rhIL-21 in NK-92 cells co-cultured with HCC SK-Hep1 cells expressed the pStat3 protein in NK cells (Fig. S4B) in addition, it significantly increased the expression of pStat3 in HCC cells (Fig. 5D). A phosphorylation of Stat3 in anticancer of NK cell is shown to be not good but is controversial yet [31]. Of note, even if the presence of rhIL-21 in their supernatants of the co-culture of HCC cells and NK cells increased the expression of pStat3 in both cells, it didn’t have the effect of the increase in HIF-1α expression in cells (Fig. 5B).

## Discussion

In this study, we first found differences in cytokine production between invasive and non-invasive HCC cells (Fig. S1). A previous study showed the high production of pro-inflammatory cytokine IL-6 in highly invasive breast cancer cell line MDA-MB-231, but not in low invasive breast cancer cell line MCF-7 [32]. Several studies have demonstrated that pro-inflammatory cytokines such as IL-1β, IL-6 and IL-8 are higher in metastatic tumors [33] and indicate poor survival in various cancer patients [34-36]. According to recent studies, IL-6 is an important cytokine in the pathogenesis of HCC [37] that is higher in the sera of HCC patients than healthy patients [8]. In humans, the presence of IL-6 in peripheral blood reduces the expression of perforin and granzyme B in NK cells, and that results in a decrease in cytotoxic ability [38]. In this regard, we thought that the presence of IL-6 in the HCC-NK interaction could decrease the activation of NK cells to HCC cells. Blocking of IL-6 in the HCC-NK interaction can change the production of cytokines. Transforming growth factor (TGF)-β significantly decreased by blocking of IL-6, while IL-18 and IL-21 extremely increased (Fig. 1). TGF-β is known to hamper the antitumor activity of NK, T and B cells in many cancers [39-41]. On the other hand, IL-18 and IL-21 contribute to the effector function of NK cells against cancers. For instance, a high dose of IL-18 significantly reduced the metastasis of tumors derived from B16F10 melanomas and CT26 colon carcinoma through an increase in PD-1 expression in splenic NK cells [42]. In addition, recent studies have shown that IL-21 can enhance the proliferation and activation of CD8^+^ T cells, and NK cell activity [30, 43]. In human NK cells, NKG2A, CD25, CD86 and CD69 were upregulated by the presence of IL-21 alone or in combination with IL-2, and that led to increased NK cytotoxicity to K562 cells [30]. The activation of STAT3 by stimulation of IL-6 and IL-8 secreted from esophageal squamous cell carcinoma (ESCC) cells downregulates the activating receptors (NKp30 and NKG2D) on the surface of NK cells [44]. In our study, blocking of IL-6 in the HCC-NK interaction did not increase cytotoxicity and activating receptors in NK cells (Figs. 2 and 3); however, the expression of caspase-3 and -9 in HCC cells significantly increased (Fig. 4). Previous studies showed that apigenin, which has an inhibitory effect on IL-6 [6, 45] induces an apoptotic effect in HCC cells through the increased expression of FasL on the surface of NK cells [46]. Blocking of IL-6 in the HCC-NK interaction upregulated the expression of FasL on the surface of NK cells (Fig. S3). Therefore, the blocking of IL-6 in the HCC-NK microenvironment might enhance the NK cytotoxicity to HCC cells by secreting granzyme B through the upregulation of FasL on the surface of NK cells.

We further identified the correlation between IL-6 and hypoxic-inducible factor HIF in the HCC-NK interaction. Hypoxia is common in tumors, which promote tumor development by restraining the various immune cells [47]. Cancers in hypoxic environment escape the immune-surveillance by a lowered activation s of immune cells [48]. For instance, immunosuppressive cells including myeloid-derived stromal cells (MDSCs), tumor-associated macrophage (TAMs), and T-regulatory (Treg) cells is commonly infiltrated to solid tumors present in hypoxic region [22]. MDSCs within the hypoxic tumors transcript HIF-1α that results in change of the function and differentiation to be more immunosuppression. The expression of HIF-1α in MDSCs ultimately increase the tolerance of T cells through the upregulation of PD-L1 [49]. According to recent reports, the transcription of HIF proteins can be increased with the stimulation of pro-inflammatory cytokines in cells under hypoxic condition. In ovarian cancer cells, the presence of IL-6 induces the expression of HIF-1α through the activation of STAT3 [28]. The pro-inflammatory cytokines are strongly upregulated by hypoxia [50]. In this study, the blocking of IL-6 in HCC-NK interaction significantly decreased the expression of HIF-1α in HCC cells (Fig. 5). The activation of HIF-1 in tumors promotes the metastasis of cancer cells by inducing the activation of oncogenic growth factors such as TGF-β3, epidermal growth factor (EGF) and others [51, 52]. NK cells within hypoxic tumors deteriorate with their cytotoxic activity and the function of activating receptors compared to the normoxic tumors. [53, 54]. The blocking of IL-6 in the interaction of HIF-1α-expressed HCC cells and NK cells enhanced the NK cytotoxicity to cancer cells and the expression of activating receptors on NK cells (Fig. 2). Of interest, the blocking of IL-6 in the HCC-NK interaction greatly increased the production of IL-21 (Fig. 1). IL-21 stimulates the effector functions of T cell or NK cell, similar to IL-2, IL-12, IL-15 and IL-18 [30, 43, 55]. NK cell induces the production of IFN-γ and the enhanced cytotoxic ability with a stimulation of IL-21 [56]. In this regard, the treatment of recombinant human (rh) IL-21 in the interaction of HIF-1α-expressed HCC cells and NK cells might increase the effector functions of NK cells. The expression of activating receptors (NKG2D, NKp44, NKp30, NKG2C) on the surface of NK cells in a co-culture of HCC cells was increased by the treatment of rhIL-21 (Fig. 6) however, that was not be better than the treatment of IL-6 antibody alone or in combination with rhIL-21 except for NKp30. The IL-21 signal generates the activation of STAT1 and STAT3 in NK cells that produce the granzyme B, perforin, and IFN-γ, and promotes the cytotoxic activity [30]. In humans, NK cells are mainly characterized by two subsets of CD56^dim^CD16^+^ and CD56^bright^CD16^dim/-^. The subset of CD56^dim^ contributes to the cytotoxic activity of NK cells, while CD56^bright^ dedicates the production of cytokines such as IFN-γ. One study shows that the activation marker of CD69 on the surface of CD56^dim^ NK cells is upregulated by the stimulation of IL-21 but not on CD56^bright^ NK cells [57]. With the stimulation of IL-21, the activation of STAT1 in NK cells regulates the natural cytotoxicity, while the activation of STAT3 in NK cells promotes the proliferative response. This is evidence that the effect of IL-21 on NK cells varies according to the activation of STAT1 and STAT3. In this study, the activation of STAT3 in NK cells was increased with the treatment of rhIL-21 but that was offset by blocking of IL-6 (Fig. S3). Of note, even if the expression of pSTAT3 in HIF-1α-expressed HCC cells co-cultured with NK cells was increased, the expression of HIF-1α in HCC cells was significantly decreased (Fig. 5). In addition, the expression of pSTAT3 in HIF-expressed HCC cells co-cultured with NK cells could be inhibited by the blocking of IL-6 in their environment. The continuous activation of STAT3 in tumors induces NK immune evasion. Targeting STAT3 in murine melanoma and leukemia models increases the immunosurveillance of NK cells. Mice with STAT3-deficient NK cells are significantly decreased in the metastasis of B16F10 melanoma [58].

Taken together, highly invasive HCC cells produce higher IL-6 than low invasive HCC cells. A high level of IL-6 in the interaction of highly invasive HCC-NK cells could diminish the NK cancer-surveillance. This study verified an increased activity of NK cells toward HCC cells through the blocking of IL-6 in HCC-NK microenvironment. In addition, the blocking effect of IL-6 in HCC-NK microenvironment was more notable in hypoxic-induced HCC cells. Of interest, IL-6 signal predominates the activation of STAT3 and HIF-1α in HCC cells even if IL-21 is present in. IL-21 is known to be cytokines stimulating the activation of NK cell however, the level of IL-6 in NK-cancer microenvironment may decide the fate of NK cells on hypoxic-induced HCC. This study might need to observe the effect of IL-21 in HCC-NK interaction. It was obvious that the blocking of IL-6 in the interaction of HIF-1α expressed HCC cells and NK cells improved the cytotoxic activity of NK cells by inducing the increased expression the activating receptors.

## Conclusion

The antitumor activity of NK cells to HIF-1α expressed HCC cells was enhanced by blocking of IL-6 in HCC-NK environment. Moreover, blocking of IL-6 increased NK cytotoxicity to HCC cells through the inhibited expression of STAT3 and HIF-1α in cancer cells.

## Supplemental Materials

Supplementary data for this paper are available on-line only at http://jmb.or.kr.

## References

[1] Yuan Y, Jiang YC, Sun CK, Chen QM (2016). Role of the tumor microenvironment in tumor progression and the clinical applications (Review). Oncol. Rep..

[2] Zhang W, Borcherding N, Kolb R (2020). IL-1 Signaling in tumor microenvironment. Adv. Exp. Med. Biol..

[3] Balkwill FR, Lee A, Aldam G, Moodie E, Thomas JA, Tavernier J (1986). Human tumor xenografts treated with recombinant human tumor necrosis factor alone or in combination with interferons. Cancer Res..

[4] Magidey-Klein K, Cooper TJ, Kveler K, Normand R, Zhang T, Timaner M (2021). IL-6 contributes to metastatic switch via the differentiation of monocytic-dendritic progenitors into prometastatic immune cells. J. ImmunoTher. Cancer.

[5] Shacter E, Arzadon GK, Williams J (1992). Elevation of interleukin-6 in response to a chronic inflammatory stimulus in mice: inhibition by indomethacin. Blood.

[6] Lee HH, Jung J, Moon A, Kang H, Cho H (2019). Antitumor and anti-invasive effect of apigenin on human breast carcinoma through suppression of IL-6 expression. Int. J. Mol. Sci..

[7] Shakiba E, Ramezani M, Sadeghi M (2018). Evaluation of serum interleukin-6 levels in hepatocellular carcinoma patients: a systematic review and meta-analysis. Clin. Exp. Hepatol..

[8] Soresi M, Giannitrapani L, D'Antona F, Florena AM, La Spada E, Terranova A (2006). Interleukin-6 and its soluble receptor in patients with liver cirrhosis and hepatocellular carcinoma. World J. Gastroenterol..

[9] Doherty DG, O'Farrelly C (2000). Innate and adaptive lymphoid cells in the human liver. Immunol. Rev..

[10] Malmberg KJ, Carlsten M, Björklund A, Sohlberg E, Bryceson YT, Ljunggren HG (2017). Natural killer cell-mediated immunosurveillance of human cancer. Semin. Immunol..

[11] Mikulak J, Bruni E, Oriolo F, Di Vito C, Mavilio D (2019). Hepatic natural killer cells: organ-specific sentinels of liver immune homeostasis and physiopathology. Front. Immunol..

[12] Lee HH, Kang H, Cho H (2017). Natural killer cells and tumor metastasis. Arch. Pharm. Res..

[13] Crane CA, Han SJ, Barry JJ, Ahn BJ, Lanier LL, Parsa AT (2010). TGF-beta downregulates the activating receptor NKG2D on NK cells and CD8^+^ T cells in glioma patients. Neuro. Oncol..

[14] Wong JL, Berk E, Edwards RP, Kalinski P (2013). IL-18-primed helper NK cells collaborate with dendritic cells to promote recruitment of effector CD8^+^ T cells to the tumor microenvironment. Cancer Res..

[15] Erler JT, Cawthorne CJ, Williams KJ, Koritzinsky M, Wouters BG, Wilson C (2004). Hypoxia-mediated down-regulation of Bid and Bax in tumors occurs via hypoxia-inducible factor 1-dependent and -independent mechanisms and contributes to drug resistance. Mol. Cell. Biol..

[16] Peng XH, Karna P, Cao Z, Jiang BH, Zhou M, Yang L (2006). Cross-talk between epidermal growth factor receptor and hypoxiainducible factor-1alpha signal pathways increases resistance to apoptosis by up-regulating survivin gene expression. J. Biol. Chem..

[17] Van Meir E (1996). Hypoxia-mediated selection of cells with diminished apoptotic potential to solid tumours. Neurosurgery.

[18] Kondo A, Safaei R, Mishima M, Niedner H, Lin X, Howell SB (2001). Hypoxia-induced enrichment and mutagenesis of cells that have lost DNA mismatch repair. Cancer Res..

[19] Dai X, Pi G, Yang SL, Chen GG, Liu LP, Dong HH (2018). Association of PD-L1 and HIF-1α coexpression with poor prognosis in *Hepatocellular Carcinoma*. Transl. Oncol..

[20] Lu Y, Hu J, Sun W, Duan X, Chen X (2015). Hypoxia-mediated immune evasion of pancreatic carcinoma cells. Mol. Med. Rep..

[21] Bettelli E, Carrier Y, Gao W, Korn T, Strom TB, Oukka M (2006). Reciprocal developmental pathways for the generation of pathogenic effector TH17 and regulatory T cells. Nature.

[22] Dang Eric V, Barbi J, Yang H-Y, Jinasena D, Yu H, Zheng Y (2011). Control of TH17/Treg balance by hypoxia-inducible factor 1. Cell.

[23] Wang L, Yi T, Zhang W, Pardoll DM, Yu H (2010). IL-17 enhances tumor development in carcinogen-induced skin cancer. Cancer Res..

[24] Chang SH, Mirabolfathinejad SG, Katta H, Cumpian AM, Gong L, Caetano MS (2014). T helper 17 cells play a critical pathogenic role in lung cancer. Proc. Natl. Acad. Sci. USA.

[25] Tobin AJ, Noel NP, Christian SL, Brown RJ (2021). Lipoprotein lipase hydrolysis products induce pro-inflammatory cytokine expression in triple-negative breast cancer cells. BMC Res. Notes.

[26] Pegram HJ, Andrews DM, Smyth MJ, Darcy PK, Kershaw MH (2011). Activating and inhibitory receptors of natural killer cells. Immunol. Cell Biol..

[27] Singh V, Khurana A, Navik U, Allawadhi P, Bharani KK, Weiskirchen R (2022). Apoptosis and pharmacological therapies for targeting thereof for cancer therapeutics. Sci.

[28] Xu S, Yu C, Ma X, Li Y, Shen Y, Chen Y (2021). IL-6 promotes nuclear translocation of HIF-1α to aggravate chemoresistance of ovarian cancer cells. Eur. J. Pharmacol..

[29] Croce M, Rigo V, Ferrini S (2015). IL-21: a pleiotropic cytokine with potential applications in oncology. J. Immunol. Res..

[30] Skak K, Frederiksen KS, Lundsgaard D (2008). Interleukin-21 activates human natural killer cells and modulates their surface receptor expression. Immunology.

[31] Gotthardt D, Sexl V (2016). STATs in NK-cells: the good, the bad, and the ugly. Front. Immunol..

[32] Lee HH, Cho H (2020). Attenuated anti-tumor activity of NK-92 cells by invasive human breast carcinoma MDA-MB-231 cells. Mol. Cell. Toxicol..

[33] Li R, Wen A, Lin J (2020). Pro-inflammatory cytokines in the formation of the pre-metastatic niche. Cancers.

[34] Heichler C, Scheibe K, Schmied A, Geppert CI, Schmid B, Wirtz S (2020). STAT3 activation through IL-6/IL-11 in cancerassociated fibroblasts promotes colorectal tumour development and correlates with poor prognosis. Gut.

[35] Silva EM, Mariano VS, Pastrez PRA, Pinto MC, Castro AG, Syrjanen KJ (2017). High systemic IL-6 is associated with worse prognosis in patients with non-small cell lung cancer. PLoS One.

[36] Zhao H, Wu L, Yan G, Chen Y, Zhou M, Wu Y (2021). Inflammation and tumor progression: signaling pathways and targeted intervention. Signal Transduct. Target. Ther..

[37] Xu J LH, Wu G, Zhu M, Li M (2021). IL-6/STAT3 Is a promising therapeutic target for hepatocellular carcinoma. Front. Oncol..

[38] Cifaldi L, Prencipe G, Caiello I, Bracaglia C, Locatelli F, De Benedetti F (2015). Inhibition of natural killer cell cytotoxicity by interleukin-6: Implications for the pathogenesis of macrophage activation syndrome. Arthritis Rheumatol..

[39] Gao Y, Souza-Fonseca-Guimaraes F, Bald T, Ng SS, Young A, Ngiow SF (2017). Tumor immunoevasion by the conversion of effector NK cells into type 1 innate lymphoid cells. Nat. Immunol..

[40] Palomares O, Martín-Fontecha M, Lauener R, Traidl-Hoffmann C, Cavkaytar O, Akdis M (2014). Regulatory T cells and immune regulation of allergic diseases: roles of IL-10 and TGF-β. Genes Immun..

[41] Jelicic K, Cimbro R, Nawaz F, Huang da W, Zheng X, Yang J (2013). The HIV-1 envelope protein gp120 impairs B cell proliferation by inducing TGF-β1 production and FcRL4 expression. Nat. Immunol..

[42] Terme M, Ullrich E, Aymeric L, Meinhardt K, Desbois M, Delahaye N (2011). IL-18 induces PD-1-dependent immunosuppression in cancer. Cancer Res..

[43] Li Y, Bleakley M, Yee C (2005). IL-21 influences the frequency, phenotype, and affinity of the antigen-specific CD8 T cell response. J. Immunol..

[44] Wu J, Gao F-x, Wang C, Qin M, Han F, Xu T (2019). IL-6 and IL-8 secreted by tumour cells impair the function of NK cells via the STAT3 pathway in oesophageal squamous cell carcinoma. J. Exper. Clin. Cancer Res..

[45] Qiu JG, Wang L, Liu WJ, Wang JF, Zhao EJ, Zhou FM (2019). Apigenin inhibits IL-6 transcription and suppresses esophageal carcinogenesis. Front. Pharmacol..

[46] Lee HH, Cho H (2022). Apigenin increases natural killer cytotoxicity to human hepatocellular carcinoma expressing HIF-1α through high interaction of CD95/CD95L. J. Microbiol. Biotechnol..

[47] Chouaib S, Noman MZ, Kosmatopoulos K, Curran MA (2017). Hypoxic stress: obstacles and opportunities for innovative immunotherapy of cancer. Oncogene..

[48] Noman MZ, Hasmim M, Messai Y, Terry S, Kieda C, Janji B (2015). Hypoxia: a key player in antitumor immune response. A review in the theme: cellular responses to hypoxia. Am. J. Physiol. Cell Physiol..

[49] Noman MZ, Desantis G, Janji B, Hasmim M, Karray S, Dessen P (2014). PD-L1 is a novel direct target of HIF-1α, and its blockade under hypoxia enhanced MDSC-mediated T cell activation. J. Exper. Med..

[50] Mancino A, Schioppa T, Larghi P, Pasqualini F, Nebuloni M, Chen I-H (2008). Divergent effects of hypoxia on dendritic cell functions. Blood.

[51] Laderoute KR, Calaoagan JM, Gustafson-Brown C, Knapp AM, Li GC, Mendonca HL (2002). The response of c-jun/AP-1 to chronic hypoxia is hypoxia-inducible factor 1 alpha dependent. Mol. Cell. Biol..

[52] Conway EM, Collen D, Carmeliet P (2001). Molecular mechanisms of blood vessel growth. Cardiovasc. Res..

[53] Balsamo M, Manzini C, Pietra G, Raggi F, Blengio F, Mingari MC (2013). Hypoxia downregulates the expression of activating receptors involved in NK-cell-mediated target cell killing without affecting ADCC. Eur. J. Immunol..

[54] Sarkar S, Germeraad WT, Rouschop KM, Steeghs EM, van Gelder M, Bos GM (2013). Hypoxia induced impairment of NK cell cytotoxicity against multiple myeloma can be overcome by IL-2 activation of the NK cells. PLoS One.

[55] Brady J, Hayakawa Y, Smyth MJ, Nutt SL (2004). IL-21 induces the functional maturation of murine NK cells. J. Immunol..

[56] de Rham C, Ferrari-Lacraz S, Jendly S, Schneiter G, Dayer J-M, Villard J (2007). The proinflammatory cytokines IL-2, IL-15 and IL-21 modulate the repertoire of mature human natural killer cell receptors. Arthritis Res. Ther..

[57] Wendt K, Wilk E, Buyny S, Schmidt RE, Jacobs R (2007). Interleukin-21 differentially affects human natural killer cell subsets. Immunology.

[58] Gotthardt D, Putz EM, Straka E, Kudweis P, Biaggio M, Poli V (2014). Loss of STAT3 in murine NK cells enhances NK celldependent tumor surveillance. Blood.

