# Supplementary material for: Role of Interleukin(IL)-6 in NK Activity to Hypoxic-Induced Highly Invasive Hepatocellular Carcinoma(HCC) Cells
Source: J Microbiol Biotechnol. 2023 Jun 12;33(7):864–74. doi: 10.4014/jmb.2304.04023 (PMC10394337; doi:10.4014/jmb.2304.04023)
Supplement: Supplementary file 1 [file jmb-33-7-864-supple.pdf]

Supplementary Figures

Role of interleukin (IL)-6 in NK activity to hypoxic-induced highly invasive hepatocellular carcinoma (HCC) cells

Hwan Hee Lee[1,2*], Hyojeung Kang[3] and Hyosun Cho[1,2*]

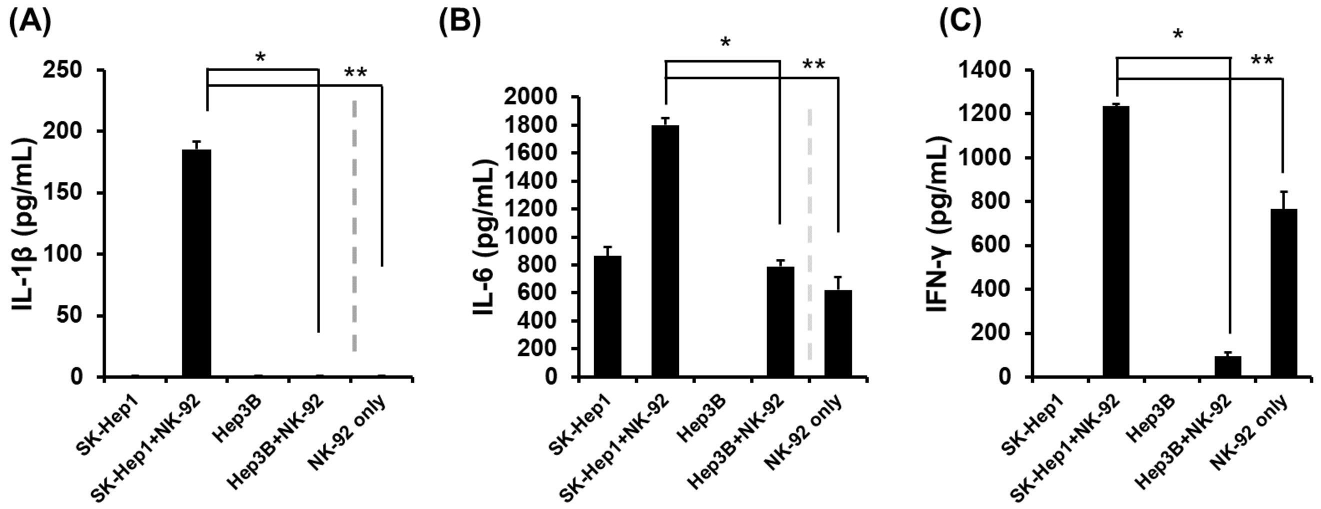

Figure S1. The production of cytokines by the interaction of human highly invasive HCC cells or low invasive HCC cells and human NK cells.

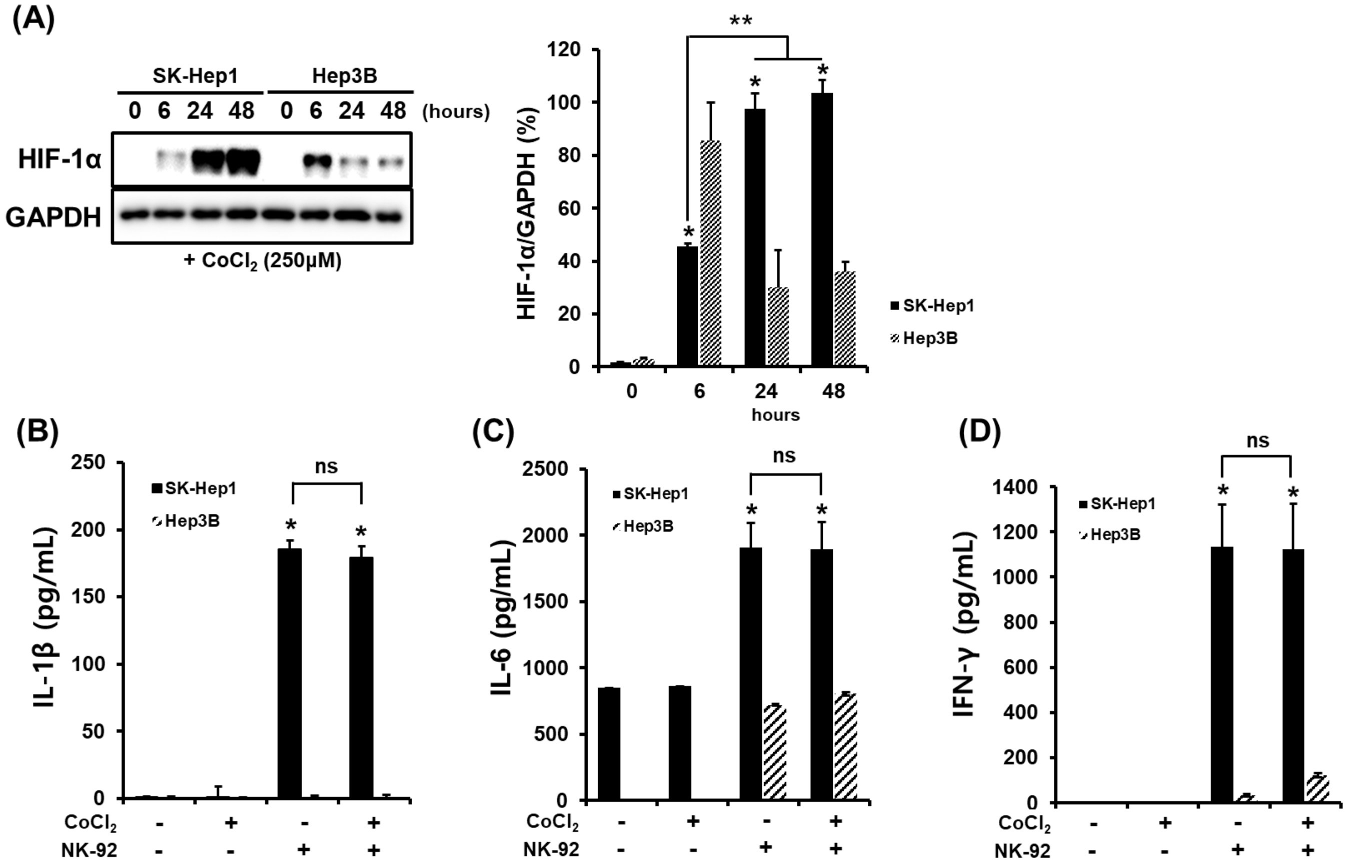

Figure S2. Pro-inflammatory cytokines produced by the interaction of highly invasive HCC cells and NK cells with a treatment of $CoCl_2$.

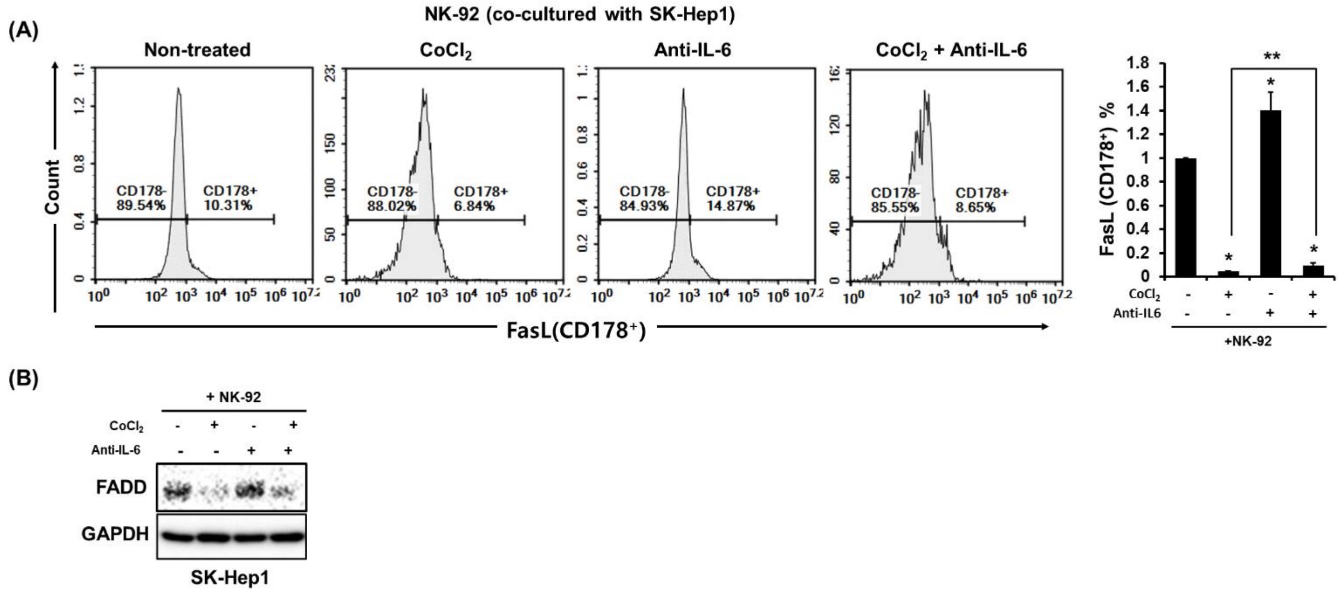

Figure S3. The expression of proteins associated with FasL(CD178)/Fas (CD95) signals in the interaction of human highly invasive HCC cells and Nk cells.

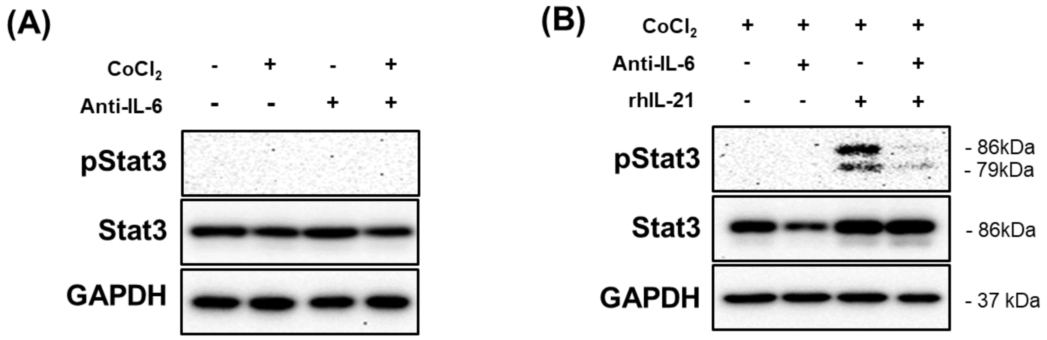

Figure S4. The expression of pStat3 in NK cells co-cultured with HIF-1α-expressed HCC cells in the presence or absence of anti-IL-6 and rhIL-21.
